# Identification of novel compound heterozygous variants in the *DNAH1* gene of a Chinese family with left-right asymmetry disorder

**DOI:** 10.3389/fmolb.2023.1190162

**Published:** 2023-06-29

**Authors:** Lamei Yuan, Xuehui Yu, Heng Xiao, Sheng Deng, Hong Xia, Hongbo Xu, Yan Yang, Hao Deng

**Affiliations:** ^1^ Health Management Center, The Third Xiangya Hospital, Central South University, Changsha, China; ^2^ Center for Experimental Medicine, The Third Xiangya Hospital, Central South University, Changsha, China; ^3^ Disease Genome Research Center, Central South University, Changsha, China; ^4^ Department of Neurology, The Third Xiangya Hospital, Central South University, Changsha, China; ^5^ Department of Pharmacy, Xiangya Hospital, Central South University, Changsha, China; ^6^ Department of Emergency, The Third Xiangya Hospital, Central South University, Changsha, China

**Keywords:** *DNAH1*, left-right asymmetry disorder, whole exome sequencing, novel variant, genetic analysis

## Abstract

Most internal organs in humans and other vertebrates exhibit striking left-right asymmetry in position and structure. Variation of normal organ positioning results in left-right asymmetry disorders and presents as internal organ reversal or randomization. Up to date, at least 82 genes have been identified as the causative genetic factors of left-right asymmetry disorders. This study sought to discover potential pathogenic variants responsible for left-right asymmetry disorder present in a Han-Chinese family using whole exome sequencing combined with Sanger sequencing. Novel compound heterozygous variants, c.5690A>G (p.Asn1897Ser) and c.7759G>A (p.Val2587Met), in the dynein axonemal heavy chain 1 gene (*DNAH1*), were found in the proband and absent in unaffected family members. Conservation analysis has shown that the variants affect evolutionarily conserved residues, which may impact the tertiary structure of the DNAH1 protein. The novel compound heterozygous variants may potentially bear responsibility for left-right asymmetry disorder, which results from a perturbation of left-right axis coordination at the earliest embryonic development stages. This study broadens the variant spectrum of left-right asymmetry disorders and may be helpful for genetic counseling and healthcare management for the diagnosed individual, and promotes a greater understanding of the pathophysiology.

## Introduction

Most human and other vertebrate internal organs asymmetrically orient along a left-right (L-R) axis and exhibit an elaborate L-R asymmetric pattern (McGrath et al., 2003; Blum et al., 2014). Genetic alterations of L-R signaling pathways may lead to L-R asymmetry disorders, which may be inherited in autosomal recessive, autosomal dominant, or X-linked modes (Deng et al., 2015; Perles et al., 2015; Grimes et al., 2016). Environmental modifiers and developmental randomness are also likely to play roles in L-R asymmetry disorders (Deng et al., 2015). Three broad types of internal organ positioning along the L-R axis are recognized (Levin, 2004; Best et al., 2019). *Situs solitus* is a condition in which all internal organs are positioned in a normal visceroatrial arrangement (Offen et al., 2016). In *situs inversus* (SI) and *heterotaxy* (HTX), there are mirror-image reversals and randomizations of visceroatrial arrangements, respectively (Offen et al., 2016; Geddes et al., 2020). SI and HTX are genetically heterogeneous disorders with reduced penetrance (Deng et al., 2015). SI with an incidence of 1 in every 8,500 live births is usually not related to congenital cardiac defects (Basu and Brueckner, 2008). Complete reversal of internal organs usually doesn’t result in discernible physiological risk, as the organs maintain their normal structures and relative positions (Bisgrove et al., 2003; Peeters and Devriendt, 2006). HTX with an incidence of 1 in 10,000 live births is related to at least 3% of all congenital cardiac disease cases (Basu and Brueckner, 2008). Other congenital anomalies in HTX usually manifest as pulmonary isomerism, intestinal malrotation, asplenia, or polysplenia (Wang et al., 2022; Wells et al., 2022).

Previously reported genetic defects implicated in L-R asymmetry disorders include complex chromosomal rearrangements, translocations, insertions/duplications, deletions, and inversions (Kosaki and Casey, 1998; Olbrich et al., 2002; Sutherland and Ware, 2009). Since the Zic family member 3 gene (*ZIC3*) variants in X-linked HTX were identified, at least 82 genes have been considered to be responsible for human L-R asymmetry disorders (Gebbia et al., 1997; Yu et al., 2022).

This study sought to identify the genetic factors responsible for the L-R asymmetry disorder present in a Han-Chinese family using whole exome sequencing (WES) combined with Sanger sequencing. Compound heterozygous variants in the dynein axonemal heavy chain 1 gene (*DNAH1*, OMIM 603332, NM_015512.5), including missense variants c.5690A>G (p.Asn1897Ser) and c.7759G>A (p.Val2587Met), may be the possible genetic etiology of the L-R asymmetry disorder. Basic Local Alignment Search Tool comparison of protein sequences revealed that p.Asn1897 and p.Val2587 were highly conserved in DNAH1 protein. Structural modeling also showed that residues are crucial to the structure or function of DNAH1 protein. These findings expand the variant spectrum of *DNAH1*, which may be beneficial for clinical and genetic diagnosis.

## Methods

### Pedigree and subjects

**FIGURE 1:**
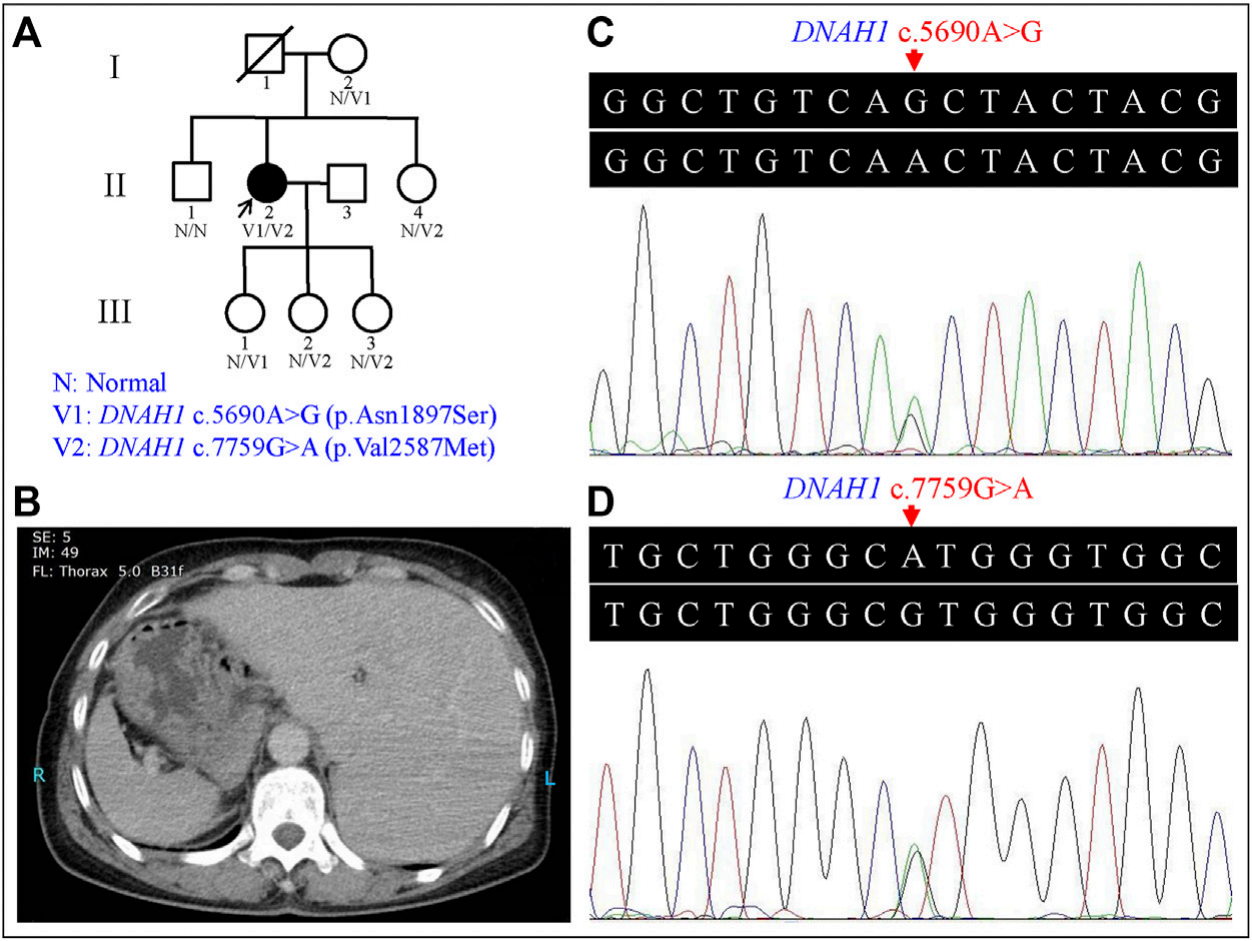
Genetic analysis of the left-right (L-R) asymmetry disorder pedigree and the representative computed tomography (CT) image of the proband. **(A)** Pedigree analysis of the L-R asymmetry disorder family. Squares and circles indicate males and females, respectively; open symbols indicate unaffected family members; the arrow indicates the proband; the symbol with a slash indicates a deceased member. **(B)** CT image of the proband showed the stomach and spleen were right-sided, and the liver was left-sided. **(C)** Heterozygous *DNAH1* c.5690A>G (p.Asn1897Ser) variant in the proband (II:2). **(D)** Heterozygous *DNAH1* c.7759G>A (p.Val2587Met) variant in the proband (II:2).

Nine members of a three-generation Han-Chinese pedigree containing the L-R asymmetry disorder sufferer were enrolled at the Third Xiangya Hospital, Central South University, P.R. China (Figure 1A). Peripheral blood samples and available clinical data were collected from the proband (II:2) and available unaffected pedigree members (I:2, II:1, II:4, III:1, III:2, and III:3). Written informed consents were given by all participants. This study was conducted in accordance with the Declaration of Helsinki and approved by the Institutional Review Board of the Third Xiangya Hospital, Central South University, Changsha, Hunan, China.

### Sample preparation and whole exome sequencing

Genomic DNA (gDNA) was isolated from peripheral blood samples according to the previously described standard method (Xiang et al., 2018; Xiao et al., 2019). WES was carried out to detect the proband’s genetic variants by BGI-Shenzhen, China (Xiao et al., 2018). Qualified gDNA was randomly broken into fragments sized from 150 bp to 250 bp. Both ends of the end-repaired DNA fragments were ligated to adapters for amplification, purification, and hybridization. Captured products were processed to form DNA nanoballs by circularization and amplification. Agilent SureSelect Human All Exon V6, which covers about 99% of the human exonic regions, was used in the exome capture. The qualified circular DNA library was loaded on a BGISEQ-500 sequencing platform to perform high-throughput sequencing (Fan et al., 2019).

### Variant analysis

Raw data (stored in FASTQ format) derived from the BGISEQ-500 sequencing platform were processed to obtain clean data. According to the strict data filtering criteria and data analysis quality control setup, the clean data were filtered from “raw data” to get access to high-quality sequencing data. Reads were cleaned during the “data cleanup” process using the following criteria: i) adapter sequence, ii) unknown base ratios more than 10%, and iii) inferior-quality base (base quality ≤5) ratios no less than 50% (Xiong et al., 2021). These clean data were mapped to the human reference genome sequence (GRCh37/hg19) using the Burrows-Wheeler Aligner (BWA, v0.7.15) software. Picard tools (v2.5.0, https://broadinstitute.github.io/picard/) were used to mark duplicated reads. Reads around insertions/deletions (indels) were realigned, and a base quality score recalibration step to improve base quality scores was conducted by Genome Analysis Toolkit (GATK, v3.3.0, https://gatk.broadinstitute.org/hc/en-us). HaplotypeCaller of GATK was applied to call a raw variant set including single nucleotide polymorphisms (SNPs) and indels. Hard-filtering methods with proper parameters were used to filter SNPs and indels (Xia et al., 2018; Xia et al., 2019). The resulting high-confident SNPs and indels were further annotated by a SnpEff tool (https://pcingola.github.io/SnpEff/). In order to find candidate variants, high-confident SNPs and indels were filtered against the 1000 Genomes Project (1000G), the National Heart, Lung, and Blood Institute (NHLBI) Exome Sequencing Project (ESP) 6500, the Exome Aggregation Consortium (ExAC), and Genome Aggregation Database (gnomAD), as well as the Single Nucleotide Polymorphism database (version 154, dbSNP154). The remaining variants with minor allele frequency (MAF) lower than 0.5% were further filtered against the BGI in-house exome databases (containing 1,943 Chinese controls without L-R asymmetry disorders).

Sanger sequencing verified the candidate variants revealed by WES in the proband and screened them in the available family members. The used primer sequences designed by Primer3 software (https://primer3.ut.ee/) for the candidate variants were 5′-TGC​CCC​TTG​GCA​TAG​AAT​AC-3′ and 5′-CAT​GGG​TGA​GGA​GGT​CAA​AC-3′, and 5′-GAA​GCT​GGT​CCT​CTT​CAT​GG-3′ and 5′-AAG​CAT​GGG​TCA​GTC​AAA​CC-3′, respectively. The detected variants were further classified according to the American College of Medical Genetics and Genomics (ACMG) guidelines for interpreting gene variants (Richards et al., 2015). Clustal Omega (http://www.ebi.ac.uk/Tools/msa/clustalo/) was used to carry out a conservative analysis by aligning nine homologous DNAH1 protein sequences retrieved from the National Center for Biotechnology Information Protein database (https://www.ncbi.nlm.nih.gov/protein/). The tertiary structures of wild-type and variant-type were conducted with the online SWISS-MODEL tool (https://swissmodel.expasy.org/) and further visualized structures were constructed via PyMOL software (version 2.3, Schrödinger, LLC, Portland, United States) (Xiang et al., 2019).

## Results

### Clinical data

The proband (II:2) is a 54-year-old female without respiratory symptoms or fertility problems. L-R asymmetry disorder was diagnosed after a routine preoperative assessment for surgical management of a multinodular goiter. From the ultrasonographic examination and computed tomography (CT) results, she was diagnosed as suffering from SI, including dextrocardia, left-sided liver, and stomach and spleen on the right side of the proband’s body (Figure 1B). Transthoracic echocardiography revealed normal characteristic morphological features and normal function of the heart, as well as normal valve morphology and function.

### Genetic analysis

Proband gDNA exome sequencing produced a total of 226.50 million clean reads. After duplicate reads removal, 201.92 million effective reads were generated. Of these, 99.94% were mapped to the human reference genome. The average sequencing depth across the target region was 249.91×, and 99.41% of the target region was covered at 10×. In total, 103,286 SNPs and 18,053 indels were detected. Commonly known variants with MAF ≥0.5% recorded in the 1000G, the NHLBI ESP6500, and the dbSNP154 databases were removed. The remaining variants were further filtered against the BGI in-house exome databases. By screening all known disease-causing genes responsible for L-R asymmetry disorders, only two compound heterozygous *DNAH1* gene missense variants, c.5690A>G (p.Asn1897Ser) in the exon 36 and c.7759G>A (p.Val2587Met) in the exon 49, were classified as potential disease-causing variants for the proband. Other potential disease-causing variants in at least 82 known genes associated with L-R asymmetry disorder phenotypes were ruled out in the proband, though large variants like complex rearrangement and gross deletion/duplication in these genes cannot be completely excluded. The c.5690A>G and c.7759G>A variants are documented in the dbSNP154 and have low frequencies in the global population (Table 1), indicating the compound heterozygous variants are potentially disorder-related variants.

**TABLE 1:** Identification of the dynein axonemal heavy chain 1 gene variants in the patient.

Variant		Variant 1	Variant 2
Nucleotide change		c.5690A>G	c.7759G>A
Amino acid change		p.Asn1897Ser	p.Val2587Met
Zygosity		Heterozygote	Heterozygote
Variant type		Missense	Missense
dbSNP154		rs138560279	rs747611842
Allelic frequencies	1000G	1.60 × 10^−3^	—
	ExAC	4.73 × 10^−4^	3.66 × 10^−4^
	gnomAD	5.06 × 10^−4^	1.86 × 10^−4^

dbSNP154, Single Nucleotide Polymorphism database (version 154); 1000G, 1000 Genomes Project; ExAC, Exome Aggregation Consortium; gnomAD, Genome Aggregation Database.

**FIGURE 2:**
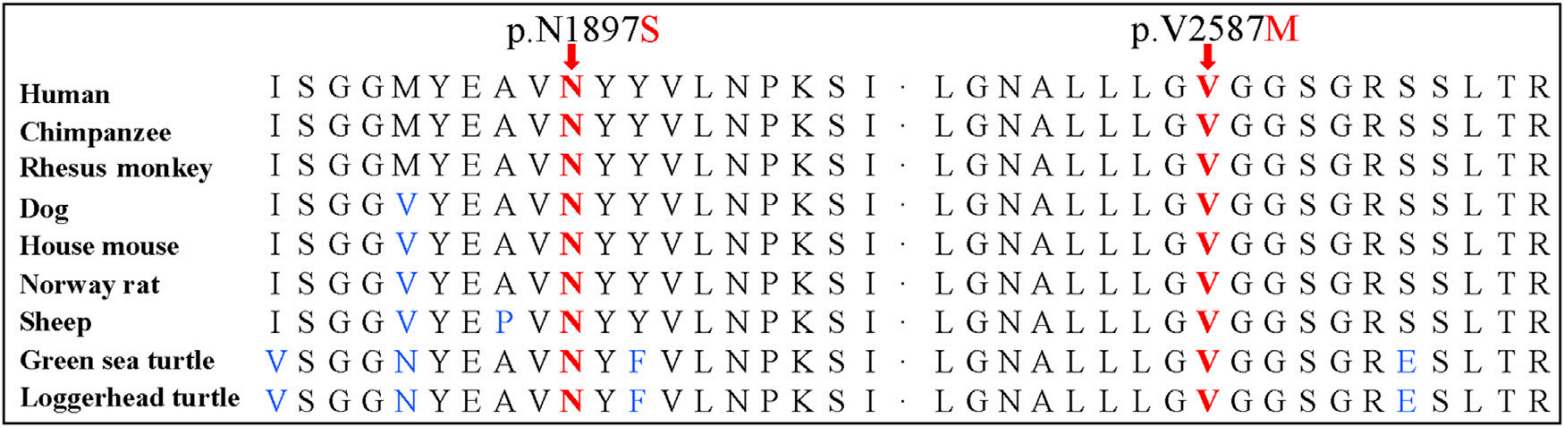
Conservation analyses of the dynein axonemal heavy chain 1 p.Asn1897 and p.Val2587 amino acid residues.

**FIGURE 3:**
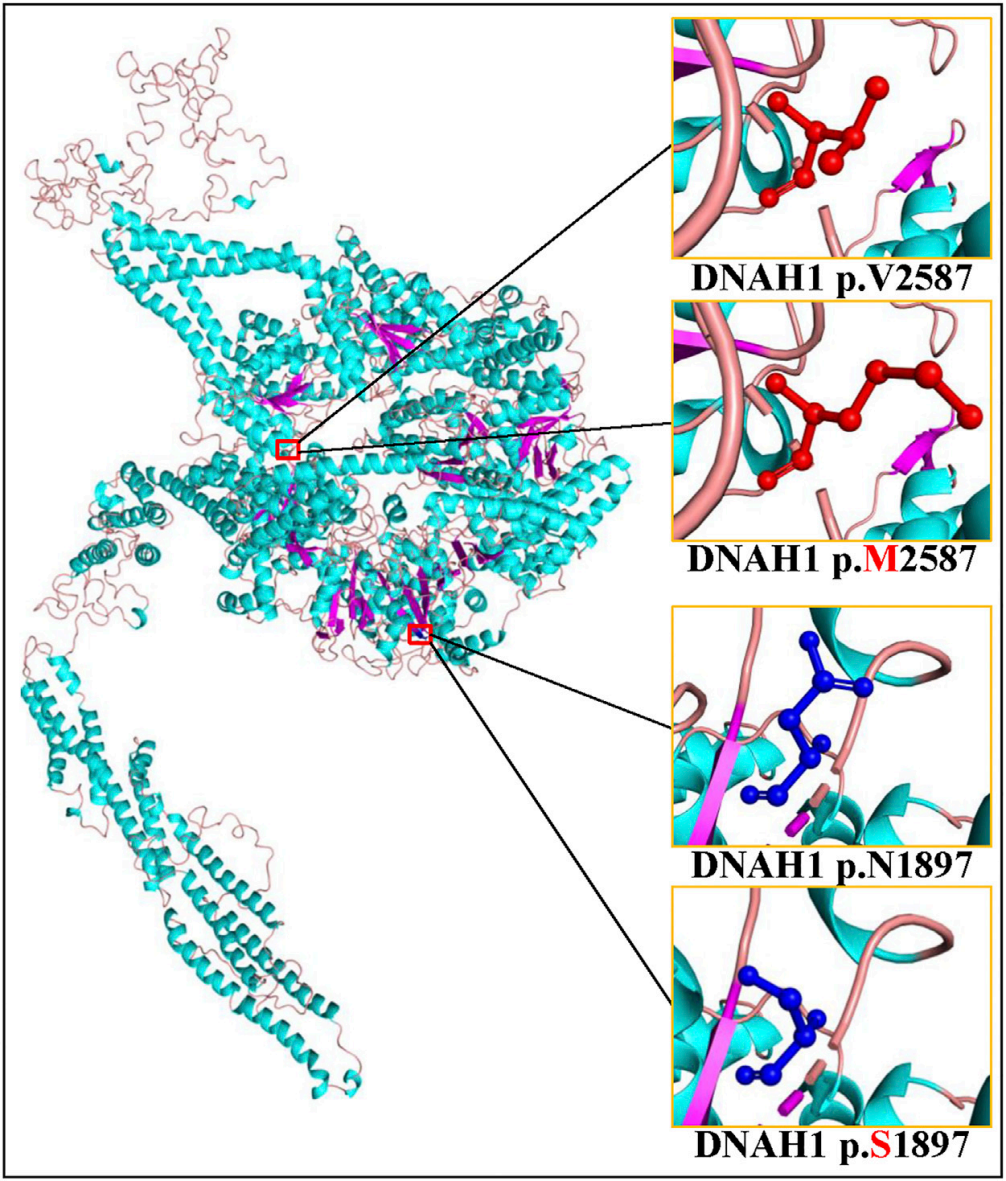
Cartoon model of the dynein axonemal heavy chain 1 (DNAH1) protein structure visualized by PyMOL based on the SWISS-MODEL. The asparagine (N) and mutated serine (S) at position 1897 and the valine (V) and mutated methionine (M) at position 2587 are indicated with ball-and-stick models.

Sanger sequencing confirmed the *DNAH1* variants c.5690A>G and c.7759G>A in the proband (Figures 1C, D). In the pedigree, unaffected family members (I:2 and III:1) had the heterozygous c.5690A>G variant, and unaffected family members (II:4, III:2, and III:3) had the heterozygous c.7759G>A variant. These results indicated that the compound heterozygous variants c.5690A>G and c.7759G>A co-segregated with L-R asymmetry disorder in the pedigree. The c.7759G>A variant was absent from 1000G and the BGI in-house exome databases. Although the c.5690A>G (rs138560279) variant was recorded in the public database, the frequencies were low, with a MAF for “G” ranging from 0.0005 (ExAC) to 0.0016 (1000G). The c.5690A>G variant was also absent from the BGI in-house exome databases. The sequence variants, c.5690A>G and c.7759G>A, were classified as “likely pathogenic” following the ACMG standards and guidelines. Clustal Omega showed that the two residues p.Asn1897 and p.Val2587 in the DNAH1 protein were fully conserved among nine vertebrates (Figure 2), indicating that the two variants are probably pathogenic. A structural model showed the conformational alterations of asparagine (Asn-1897) into serine (Ser-1897) and valine (Val-2587) into methionine (Met-2587), further supporting the possible pathogenicity of the variants (Figure 3).

## Discussion

Human L-R asymmetry disorders may arise as a manifestation of a wide disease spectrum, such as primary ciliary dyskinesia (PCD), polycystic kidney disease 2 (PKD2), nephronophthisis, and Bardet-Biedl syndrome (Kosaki and Casey, 1998; Bataille et al., 2011; Marion et al., 2012). SI may be an incidental discovery in asymptomatic individuals during health examinations since complete mirror-image reversal of all asymmetrical structures may pose no detriment (Casey, 1998; Bisgrove et al., 2003). L-R asymmetry establishment in vertebrates generally has four phases: L-R symmetry breaking at early embryonic stages, transfer of asymmetric signals, asymmetric expression of left determinants in the left lateral plate mesoderm, and visceral organ L-R asymmetric morphogenesis (Gebbia et al., 1997; Okada et al., 2005; Shiraishi and Ichikawa, 2012). The genes encoding dynein axonemal heavy chains (DNAHs), such as *DNAH5* (OMIM 603335), *DNAH6* (OMIM 603336), *DNAH9* (OMIM 603330), *DNAH11* (OMIM 603339), and *DNAH17* (OMIM 610063), are involved in L-R symmetry disorder development. Genetic variants in the above human *DNAH* genes have been reported to be associated with cilia and flagella dysfunction (Fliegauf et al., 2005; Hornef et al., 2006; Li et al., 2016; Xiong et al., 2021; Yu et al., 2022).

The *DNAH1* gene, located on 3p21.1, comprises 78 exons and encodes an inner dynein arm heavy chain with 4,265 amino-acid residues. The DNAH1 protein functions as an ATP-dependent motor protein that generates force towards the minus end of microtubules, which is required for the bending of cilia and sperm flagellum motility (McGrath and Brueckner, 2003; Wambergue et al., 2016). Its C-terminal ∼3,500 amino-acid residues constitute the conserved dynein motor domain, which conducts the ATP-hydrolysis process in a ring of AAA+ (extended ATPases associated with various cellular activities) domains (Wendler et al., 2012; Schmidt and Carter, 2016). Dyneins play roles in mitosis, cellular transport, ciliary and flagellar motility, and L-R asymmetry determination (Supp et al., 1997; Supp et al., 1999; Basu and Brueckner, 2008; Schmidt et al., 2015). DNAH1 protein deficiencies may result in ciliary structure and function defects and impair sperm axoneme biogenesis, proposed to result in PCD and male infertility, respectively (McGrath and Brueckner, 2003; Imtiaz et al., 2015; Yang et al., 2018). Previously identified *DNAH1* gene variants include at least 29 missense variants, 11 nonsense variants, 11 splicing variants, 6 frameshift variants, a deletion variant, and an extension variant (Table 2). Except for the *DNAH1* gene homozygous missense variant c.3460A>C (p.Lys1154Gln) that was reported to be responsible for PCD in combination with SI (Imtiaz et al., 2015), the other known variants were involved in male infertility (Amiri-Yekta et al., 2016; Sha et al., 2017; Wang et al., 2017). Strikingly, the proband in our study denied the history of *in vitro* fertilization, and seems to have three biological children who inherited the possible disease-causing variants c.5690A>G (III:1) and c.7759G>A (III:2 and III:3), consistent with the reports that variants in *DNAH* genes may also not cause infertility (Xiong et al., 2021; Feng et al., 2022; Yu et al., 2022).

**TABLE 2:** Clinical data of the dynein axonemal heavy chain 1 gene variant carriers in different families.

Ped	Case	Sex			Age			GT				Nucleotide change			Amino acid change	Variant type			IF			PCD			Situs		References		
P1	NA	NA			NA			CH				c.1286+7C>A, c.5356C>T			NA, p.R1786C	Splicing, missense			NA			Y			N		Guan et al. (2021)		
P2	II:1	M			34 years			CH				c.1336G>C, c.2912G>A			p.E446Q, p.R971H	Missense, missense			Y			N			NA		Hu et al. (2021)		
P3	NA	M			32 years			CH				c.2602C>T, c.12748C>T			p.R868*, p.R4250*	Nonsense, nonsense			Y			N			N		Yu et al. (2021)		
P4	NA	NA			NA			Hom				c.2610G>A			p.W870*	Nonsense			NA			Y			N		Guan et al. (2021)		
P5	NA	M			32 years			CH				c.2610G>A, c.12287G>T			p.W870*, p.R4096L	Nonsense, missense			Y			N			NA		Sha et al. (2017)		
P6	NA	NA			NA			CH				c.2912G>A, c.11135G>A			p.R971H, p.R3712Q	Missense, missense			NA			Y			N		Guan et al. (2021)		
P7	NA	M			32 years			CH				c.3108G>A, c.5864G>A			p.W1036*, p.W1955*	Nonsense, nonsense			Y			N			NA		Sha et al. (2017)		
P8	III:1	F			NA			Hom				c.3460A>C			p.K1154Q	Missense			Y			Y			SI		Imtiaz et al. (2015)		
	III:2	F			NA			Hom				c.3460A>C			p.K1154Q	Missense			Y			Y			SI				
P9	NA	NA			NA			CH				c.3836A>G, c.6328_6337del			p.K1279R, p.S2110Gfs*19	Missense, frameshift			NA			Y			N		Guan et al. (2021)		
P10	NA	M			31 years			CH				c.3836A>G, c.11726_11727del			p.K1279R, p.P3909Rfs*33	Missense, frameshift			Y			N			NA		Sha et al. (2017)		
P11	III:1	M			NA			Hom				c.3860T>G			p.V1287G	Missense			Y			N			NA		Amiri-Yekta et al. (2016)		
	III:3	M			NA			Hom				c.3860T>G			p.V1287G	Missense			Y			N			NA				
P12	NA	M			NA			Hom				c.3877G>A			p.D1293N	Missense			Y			N			NA		Ben Khelifa et al. (2014)		
P13	NA	M			32 years			CH				c.4115C>T, c.11726_11727del			p.T1372M, p.P3909Rfs*33	Missense, frameshift			Y			N			NA		Sha et al. (2017)		
P14	NA	M			29 years			CH				c.4552C>T, c.9685C>T			p.Q1518*, p.R3229C	Nonsense, missense			Y			NA			NA		Yu et al. (2021)		
P15	NA	M			28 years			CH				c.4552C>T, c.11787+1G>A			p.Q1518*, NA	Nonsense, splicing			Y			NA			NA		Yu et al. (2021)		
P16	NA	M			41 years			CH				c.4552C>T, c.12287G>T			p.Q1518*, p.R4096L	Nonsense, missense			Y			NA			NA		Yu et al. (2021)		
P17	IV:1	M			28 years			CH				c.4670C>T, c.8170C>T			p.T1557M, p.R2724*	Missense, nonsense			Y			NA			NA		Jiang et al. (2021)		
P18	NA	M			NA			Hom				c.5094+1G>A			NA	Splicing			Y			N			NA		Ben Khelifa et al. (2014)		
P19	NA	M			24 years			CH				c.5104C>T, c.11726_11727del			p.R1702*, p.P3909Rfs*33	Nonsense, frameshift			Y			NA			NA		Yu et al. (2021)		
P20	NA	M			NA			CH				c.5105G>A, c.10823+1G>C			p.R1702Q, NA	Missense, splicing			Y			NA			NA		Oud et al. (2021)		
P21	NA	M			28 years			CH				c.5573T>C, c.11726_11727del			p.L1858P, p.P3909Rfs*33	Missense, frameshift			Y			NA			NA		Yu et al. (2021)		
P22	NA	M			22 years			CH				c.5626G>C, c.7066C>T			p.A1876P, p.R2356W	Missense, missense			Y			NA			NA		Yang et al. (2018)		
P23	II:2	F			54 years			CH				c.5690A>G, c.7759G>A			p.N1897S, p.V2587M	Missense, missense			N			N			SI		This study		
P24	NA	M			30 years			CH				c.5766–2A>G, c.10630G>T			NA, p.E3544*	Splicing, nonsense			Y			N			NA		Sha et al. (2017)		
P25	NA	M			27 years			CH				c.6004C>T, c.10982C>A			p.R2002C, p.S3661*	Missense, nonsense			Y			NA			NA		Yu et al. (2021)		
P26	NA	M			NA			CH				c.6212T>G, c.12200_12202del			p.L2071R, p.N4069del	Missense, deletion			Y			N			NA		Sha et al. (2017)		
P27	NA	M			35 years			CH				c.6253_6254del, c.11726_11727del			p.E2086Gfs*8, p.P3909Rfs*33	Frameshift, frameshift			Y			N			NA		Sha et al. (2017)		
P28	NA	M			33 years			Het				c.6446T>G			p.L2149R	Missense			Y			NA			NA		Yang et al. (2018)		
P29	NA	M			30 years			CH				c.6526–1G>T, c.9850G>A			NA, p.E3284K	Splicing, missense			Y			NA			NA		Yu et al. (2021)		
P30	NA	M			42 years			CH				c.6822C>G, c.9850G>A			p.D2274E, p.E3284K	Missense, missense			Y			N			NA		Sha et al. (2017)		
P31	NA	M			43 years			CH				c.6912C>A, c.7076G>T			p.R2304*, p.R2359L	Nonsense, missense			Y			N			NA		Zhuang et al. (2022)		
P32	II:1	M			36 years			CH				c.7066C>T, c.11726_11727del			p.R2356W, p.P3909Rfs*33	Missense, frameshift			Y			N			NA		Sha et al. (2017)		
	II:3	M			31 years			CH				c.7066C>T, c.11726_11727del			p.R2356W, p.P3909Rfs*33	Missense, frameshift			Y			N			NA				
P33	NA	M			31 years			CH				c.7201del, c.7205C>A			p.A2402Pfs*39, p.A2402D	Frameshift, missense			Y			NA			NA		Yang et al. (2018)		
P34	NA	M			28 years			Hom				c.7377+1G>C			NA	Splicing			Y			N			NA		Sha et al. (2017)		
P35	NA	M			33 years			CH				c.7397G>A, c.12287G>A			p.R2466Q, p.R4096H	Missense, missense			Y			NA			NA		Yu et al. (2021)		
P36	NA	F			15 years			Het				c.7795G>T			p.A2599S	Missense			NA			Y			N		Emiralioğlu et al. (2020)		
P37	NA	M			22 years			CH				c.8322+3del, c.11726_11727del			NA, p.P3909Rfs*33	Splicing, frameshift			Y			NA			NA		Yang et al. (2018)		
P38	NA	M			NA			Hom				c.8626–1G>A			NA	Splicing			Y			N			NA		Amiri-Yekta et al. (2016)		
	NA	M			NA			Hom				c.8626–1G>A			NA	Splicing			Y			N			NA				
	NA	M			NA			Hom				c.8626–1G>A			NA	Splicing			Y			N			NA				
P39	NA	M			NA			Hom				c.8626–1G>A			NA	Splicing			Y			N			NA		Amiri-Yekta et al. (2016)		
P40	NA	M			25 years			Het				c.11412del			p.L3805Sfs*7	Frameshift			Y			NA			NA		Yang et al. (2018)		
P41	NA	M			40 years			Hom				c.11726_11727del			p.P3909Rfs*33	Frameshift			Y			N			NA		Wang et al. (2017)		
P42	NA	M			38 years			Hom				c.11726_11727del			p.P3909Rfs*33	Frameshift			Y			N			NA		Wang et al. (2017)		
	NA	M			37 years			Hom				c.11726_11727del			p.P3909Rfs*33	Frameshift			Y			N			NA				
P43	NA	M			33 years			Hom				c.11726_11727del			p.P3909Rfs*33	Frameshift			Y			N			NA		Wang et al. (2017)		
P44	II:1	F			31 years			Hom				c.11726_11727del			p.P3909Rfs*33	Frameshift			Y			N			NA		Liu et al. (2021)		
P45	NA	M			31 years			Het				c.11726_11727del			p.P3909Rfs*33	Frameshift			Y			NA			NA		Yang et al. (2018)		
P46	NA	M			32 years			Hom				c.11726_11727del			p.P3909Rfs*33	Frameshift			Y			NA			NA		Yu et al. (2021)		
P47	NA	M			27 years			CH				c.11726_11727del, c.12089+1G>A			p.P3909Rfs*33, NA	Frameshift, splicing			Y			NA			NA		Yu et al. (2021)		
P48	NA	M			25 years			CH				c.11726_11727del, c.12264_12265del			p.P3909Rfs*33, p.W4089Gfs*51	Frameshift, frameshift			Y			NA			NA		Yu et al. (2021)		
P49	NA	M			40 years			CH				c.11726_11727del, c.12397C>T			p.P3909Rfs*33, p.R4133C	Frameshift, missense			Y			N			NA		Sha et al. (2017)		
P50	NA	M			NA			Hom				c.11788–1G>A			NA	Splicing			Y			N			NA		Ben Khelifa et al. (2014)		
P51	NA	M			NA			Hom				c.11788–1G>A			NA	Splicing			Y			N			NA		Ben Khelifa et al. (2014)		
	NA	M			NA			Hom				c.11788–1G>A			NA	Splicing			Y			N			NA				
	NA	M			NA			Hom				c.11788–1G>A			NA	Splicing			Y			N			NA				
P52	NA	M			NA			Hom				c.12796T>C			p.*4266Qext*?	Extension			Y			N			NA		Ben Khelifa et al. (2014)		

Ped, pedigree number; NA, not available; M, male; F, female; GT, genotype; CH, compound heterozygote; Hom, homozygote; Het, heterozygote; IF, infertility; Y, yes; N, no; PCD, primary ciliary dyskinesia; SI, *situs inversus*.

In this study, two *DNAH1* gene variants were identified in a Han-Chinese family including L-R asymmetry disorder sufferer. The *DNAH1* variants c.5690A>G and c.7759G>A are located at AAA2 and a highly conserved nucleotide-binding motif (P-loop) in AAA4 (UniProt ID Q9P2D7), respectively (Mocz and Gibbons, 2001; Kon et al., 2004). These two *DNAH1* variants may interfere with the rigid block formed by the whole AAA2-AAA4 region which may produce detrimental effects on the inner dynein arm heavy chains involved in generating oscillating beating in cilia (Shingyoji et al., 1998; Mocz and Gibbons, 2001; Schmidt et al., 2015). Perturbation may occur at the earliest stages in the signaling pathways that coordinate the L-R asymmetry and result in deficient embryonic nodal flow, impaired asymmetric transport of L-R signals and gene expression, and the final complete inversion of the L-R axis (Supp et al., 1997; Bisgrove et al., 2003; Peeters and Devriendt, 2006). PCD is a genetically and clinically heterogeneous disease with a diverse phenotype spectrum including chronic respiratory tract infections, L-R asymmetry disorders, and infertility (Lobo et al., 2015; Horani and Ferkol, 2018). Approximately half of PCD patients had SI or HTX (Basu and Brueckner, 2008; Deng et al., 2015). The proband in this study did not have related respiratory symptoms or fertility problems and cannot be diagnosed as typical PCD. The lack of respiratory symptoms may be due to the underlying compensation role of other dyneins that are phylogenetically close to *DNAH1*, such as *DNAH3* (OMIM 603334), *DNAH7* (OMIM 610061), and *DNAH12* (OMIM 603340) (Ben Khelifa et al., 2014). Similarly, biallelic *DNAH17* carriers and a few *DNAH9* patients were reported to only exhibit SI or sperm flagellum defects, but without other cilia-related symptoms (Fliegauf et al., 2005; Yu et al., 2022). Our patient only has L-R asymmetry disorder, without other PCD-associated disorders, which may also be counted as a variant form of PCD suffering a mild consequence of cilia dysfunction. A limitation of this study is the lack of nasal epithelial brush biopsy samples for cilia beat and ultrastructure analysis.

Cilia are central to the initial breaking of L-R symmetry (Basu and Brueckner, 2008; Zhu et al., 2020; Little and Norris, 2021). During the development of vertebrate L-R asymmetry, motile embryonic cilia produce leftward extracellular fluid flow to initiate the event that converts early embryonic bilateral symmetry to a left-sided heart and asymmetric arrangement of visceral organs (Brody, 2004; Fliegauf et al., 2005). Structural and functional ciliary defects are related to hydrocephalus, Kartagener’s syndrome, infertility, PKD2, and randomization of the L-R axis (Ibañez—Tallon et al., 2002; Brody, 2004; Fliegauf et al., 2005). More than 100 genes may be involved in L-R asymmetry defects in model organisms (Catana and Apostu, 2017). *Dnah5* mutations in mouse models result in the randomization of visceral organs’ laterality (Ibañez—Tallon et al., 2002; Olbrich et al., 2002). In *Dnah1* mutant mice, abnormal sperm behavior, fertilization failure, and reduced ciliary beat frequency were observed, similar to phenotypes of patients suffering from infertility and PCD (Neesen et al., 2001; Hu et al., 2019). The identification of the *DNAH1* gene variants in PCD patients with SI and the limited reports of animal models implies that more cases and animal models are warranted to fully reveal the effect of the *DNAH1* gene variants on L-R asymmetry (Neesen et al., 2001; Imtiaz et al., 2015).

Early L-R asymmetry disorder diagnosis may be beneficial to patients when they need external chest compression or emergency surgery for heart attack or abdominal trauma. Plain chest radiographs, echocardiography, abdominal sonography, CT, and magnetic resonance imaging are effective means of discovering and diagnosing L-R asymmetry disorders (Winer-Muram, 1995).

In summary, the novel compound heterozygous *DNAH1* gene c.5690A>G (p.Asn1897Ser) and c.7759G>A (p.Val2587Met) variants were identified in a Han-Chinese pedigree containing L-R asymmetry disorder sufferer. We present, for the first time, evidence that *DNAH1* variants do not necessarily lead to female infertility. This conclusion is based on our analysis of the female proband and her female offsprings, and further discovery of more such cases, especially homozygous variants cases, may help to understand the genotype-phenotype association of *DNAH1*. The discovery provides new evidence of the potential association between the *DNAH1* gene and L-R asymmetry disorders and extends the phenotypic spectrum of *DNAH1*-associated diseases. It supports the notion that laterality disorders may result from disturbances at the primary cilia level (Peeters and Devriendt, 2006). This work may promote a better understanding of the genetic causes underlying L-R asymmetry disorders and assist in genetic counseling and management of diagnosed individuals.

## Data Availability

The datasets presented in this study can be found in online repositories. The names of the repository/repositories and accession number(s) can be found below: https://db.cngb.org/, CNP0003867
